# Co-Culture of *Auxenochlorella protothecoides* and *Serratia liquefaciens* Promotes Lutein Accumulation

**DOI:** 10.3390/md23090360

**Published:** 2025-09-18

**Authors:** Weiwei Xue, Zhen Li, Yanhong Qiu, Yong Ma, Yongchang Xue, Zongshen Zhang, Changbin Liu

**Affiliations:** 1 School of Biological Engineering, Dalian Polytechnic University, Dalian 116034, China; 2 Guangdong Celconta Biotechnology Co., Ltd., Dongguan 523808, China

**Keywords:** *Auxenochlorella protothecoides*, *Serratia liquefaciens*, co-culture, lutein

## Abstract

Lutein, a crucial carotenoid with diverse biological roles, is in high demand in the market. Current production predominantly relies on plant extraction, which is hindered by low yield and seasonal limitations. Microalgae, such as Chlorella and Chlamydomonas, known for their efficient lutein production due to high photosynthetic efficiency, rapid growth, and ease of cultivation, still require enhanced yields. This study presents a novel finding that co-cultivating *A. protothecoides* with *S. liquefaciens* significantly boosts lutein production. Optimization of carbon and nitrogen sources, nitrogen-to-phosphorus (N:P) ratio, and algal-bacterial inoculation ratio using BG11 medium was systematically conducted. The results indicate that supplementing with 3.0 g/L sodium acetate as the carbon source, 2.0 g/L sodium nitrate as the nitrogen source, sodium dihydrogen phosphate to achieve an N:P ratio of 12:1, and an algal:bacterial inoculation ratio of 10:1, resulted in an *A. protothecoides* biomass of 21.72 g/L (DWt) and a lutein yield significantly increased to 56.86 mg/g (DWt), a ninefold rise compared to monoculture. This co-cultivation approach offers a promising avenue for sustainable industrial lutein production.

## 1. Introduction

Lutein, a prominent carotenoid, has attracted considerable interest for its established effectiveness in mitigating age-related macular degeneration and promoting visual health [1]. Functioning as a potent natural antioxidant, lutein efficiently eliminates free radicals and alleviates oxidative stress, thereby potentially contributing to the prevention and treatment of diverse chronic ailments such as cardiovascular diseases, cancer, and neurodegenerative disorders [2]. In addition to its health-related advantages, lutein finds extensive application in the food sector as a natural coloring agent, lending yellow to orange tones to various food items [3].

Lutein production is predominantly dependent on extracting it from marigold petals, a method fraught with challenges such as seasonal limitations, elevated expenses, and suboptimal extraction efficacy [4]. As a result, there is a pressing need to explore alternative, sustainable, and more effective approaches to lutein production. In recent years, rapid progress in microalgal biotechnology has positioned microalgae as a promising source for lutein production, attracting increasing research interest [5]. Microalgae offer advantages such as fast growth rates, high photosynthetic efficiency, and ease of cultivation, facilitating significant lutein accumulation in short periods [6,7]. Specific green algae species, such as *Chlorella vulgaris* (*C. vulgaris*) [8], *Chlorella sorokiniana* (*C. sorokiniana*) [9], *Phaeodactylum tricornutum* [10], and *Chlamydomonas reinhardtii* [11], have been recognized for their efficient lutein production. Among these, *Auxenochlorella protothecoides* (*A. protothecoides*) is notable for its high lutein content and strong heterotrophic growth capacity [12,13]. Studies suggest that optimizing cultivation conditions and employing genetic engineering techniques can substantially increase lutein yield in *A. protothecoides* [14]. Moreover, the co-cultivation of microalgae and bacteria offers a new approach for enhancing lutein production efficiency [15,16,17]. By harnessing synergistic microbial interactions, these systems enhance the utilization of nutrients, leading to higher yields of the desired metabolite.

Research on microalgal-bacterial co-cultures has recently gained momentum. Makaranga and Jutur demonstrated that co-culturing *Chlorella saccharophila* UTEX247 with the symbiotic bacterium *Exiguobacterium* sp. notably increased lutein yield while maintaining biomass accumulation [18]. This strategy enhances lutein biosynthesis efficiency, system stability, and adaptability by modulating metabolic activities within the microbial community [19]. Despite advancements, the utilization of microalgal-bacterial co-cultures for lutein production is still in its early stages, facing challenges such as managing microbial competition, regulating metabolic byproduct exchanges, and optimizing cultivation parameters. Therefore, understanding the intricate interaction mechanisms between microalgae and bacteria and refining co-culture conditions are crucial for enhancing lutein yields and enabling large-scale production [17,19]. This study aims to establish a high-performance co-culture system by screening compatible microalgal and bacterial strains to explore their potential for efficient lutein biosynthesis. We will systematically investigate the influence of co-cultivation parameters on lutein synthesis, laying a theoretical foundation and offering technical support for sustainable lutein production.

## 2. Results

### 2.1. Screening of Bacterial Strains

**Figure 1:**
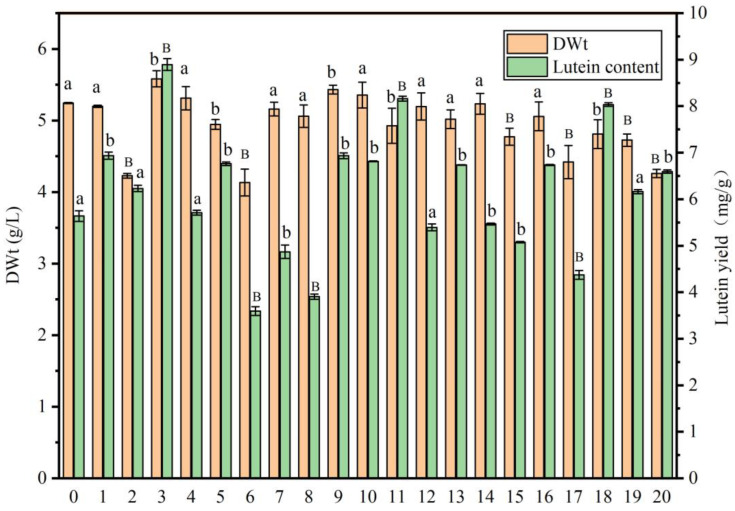
The impact of various bacterial strains on both biomass production and lutein accumulation in *A. protothecoides.* Strain 0 represents the axenic culture of *A. protothecoides*, while strains 1 to 20 denote the co-cultures of 20 different bacterial strains with *A. protothecoides*. Identical letters indicate no significant difference between groups (*p* > 0.05), while different letters signify significant differences (lowercase letters: *p* < 0.05; uppercase letters: *p* < 0.01).

Twenty bacterial strains were randomly selected from activated sludge sourced from a municipal wastewater treatment plant and co-cultured with *A. protothecoides* for 10 days. Following this, the biomass and lutein content of the alga were quantified, with the outcomes depicted in Figure 1. Notably, Strains 3 and 9 were found to significantly increase the dry weight of *A. protothecoides* (*p* < 0.05), while the lutein content in groups 3, 11, and 18 was significantly higher than that in the control group (*p* < 0.01). As a result, co-cultivation of Strain 3 with *A. protothecoides* significantly increases biomass (*p* < 0.05) and markedly enhances the lutein content in algal cells (*p* < 0.01). Therefore, Strain 3 was chosen for further taxonomic classification and investigations into algal-bacterial co-cultures.

### 2.2. Strain Characterization and Identification

**Figure 2:**
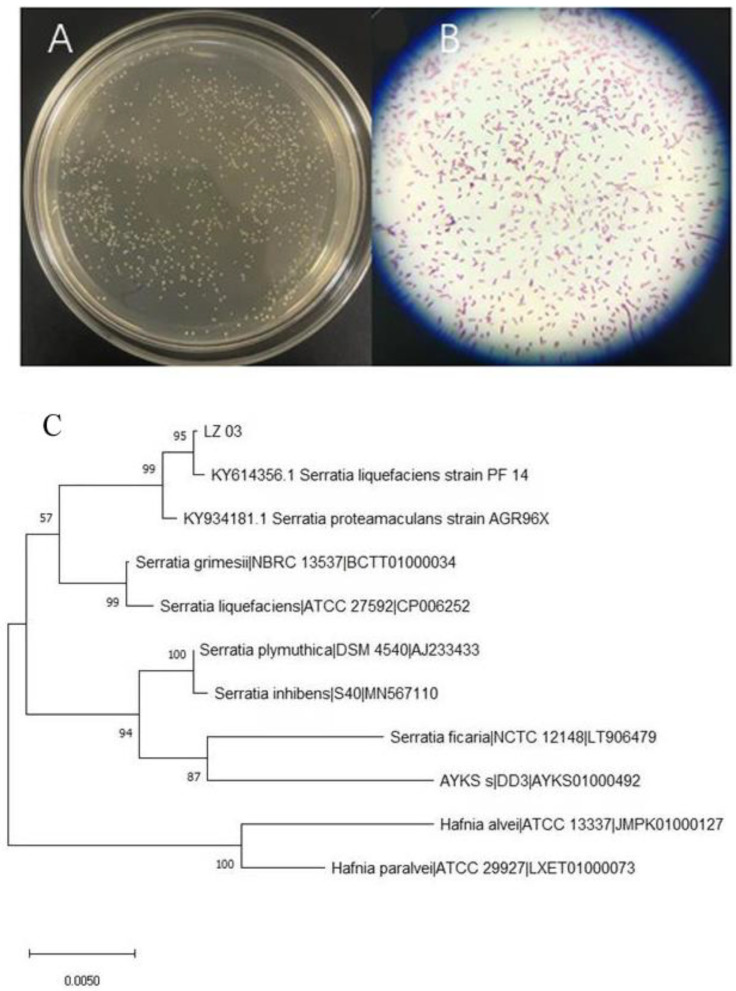
The characterization and identification of *S. liquefaciens* LZ03. (**A**) The morphological characteristics of colonies on LB agar. (**B**) Gram staining, revealing Gram-negative, rod-shaped cells. (**C**) Phylogenetic analysis utilizing the 16S rRNA gene sequence, with a Maximum Likelihood tree constructed using MEGA7 software.

Strain 3 was cultivated on fresh LB agar, yielding circular colonies with smooth margins, a convex shape, powdery texture, and creamy-white pigmentation (Figure 2A). Microscopic examination following Gram staining displayed red-colored, short rod-shaped cells, confirming its Gram-negative classification (Figure 2B). Genomic DNA was then isolated from Strain 3, and the 16S rRNA gene was PCR amplified using universal primers, sequenced, and subjected to BLAST (blastn) analysis against the NCBI database. Sequence comparison indicated a 99% similarity to *Serratia. liquefaciens* (*S. liquefaciens*) strain PF 14. Phylogenetic analysis conducted using MEGA7 software (Figure 2C) further substantiated this taxonomical assignment. Therefore, through a comprehensive assessment encompassing morphological attributes, Gram staining characteristics, and 16S rRNA gene sequence analysis, the strain was conclusively identified as *S. liquefaciens*, and subsequently denoted as *S. liquefaciens* LZ03.

### 2.3. Growth Analysis of S. liquefaciens LZ03

**Figure 3:**
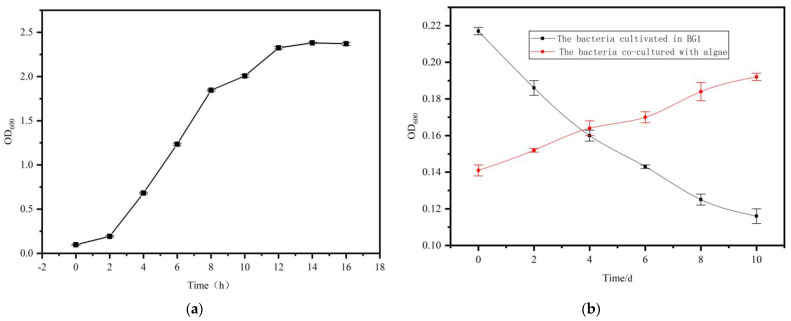
Growth dynamics of *S. liquefaciens* LZ03. (**a**) Growth curve of *S. liquefaciens* LZ03 in LB medium. (**b**) The comparison of growth between axenic culture of *S. liquefaciens* and co-culture with *A. protothecoides* in BG11 medium.

A sole colony of *S. liquefaciens* LZ03 was introduced into sterile LB medium and incubated at 28 °C for 16 h. OD_600_ was monitored every 2 h to construct the growth curve depicted in Figure 3a. The growth curve demonstrates that *S. liquefaciens* LZ03 transitioned into the logarithmic growth phase within 2 h of incubation and attained the stationary phase by the 12th h.

To evaluate the proliferation of *S. liquefaciens* LZ03 in BG11 medium, we inoculated the strain individually or in conjunction with *A. protothecoides* at a ratio of 10:1 (*v*/*v*) in sterile BG11 liquid medium. The cultures were incubated for a period of 10 days under both axenic and co-culture conditions. The findings reveal contrasting growth trajectories (Figure 3b). Specifically, while the biomass of *S. liquefaciens* LZ03 declined steadily when cultivated alone in BG11 medium, it exhibited a progressive increase when co-cultured with *A. protothecoides*. This observed augmented growth of *S. liquefaciens* LZ03 in co-culture implies a probable provision of vital nutrients by *A. protothecoides* to support bacterial expansion.

### 2.4. Effect of Algal:Bacterial Ratio on Growth and Lutein Accumulation in A. protothecoides Co-Cultures

**Figure 4:**
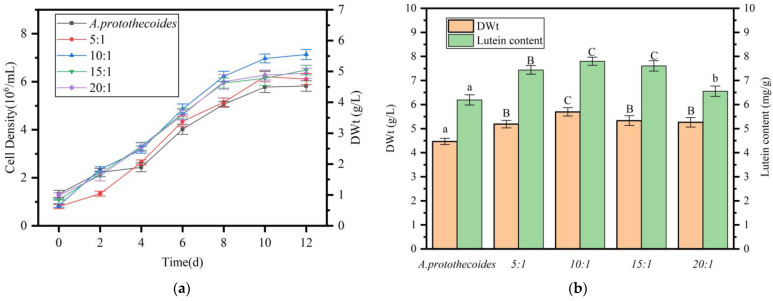
Impact of *S. liquefaciens* LZ03 inoculum size on *A. protothecoides* performance in co-culture. (**a**) Growth kinetics of *A. protothecoides* under varying bacterial inoculum ratios. (**b**) Final biomass (DWt) and lutein content of *A. protothecoides* co-cultures. Identical letters indicate no significant difference between groups (*p* > 0.05), while different letters signify significant differences (lowercase letters: *p* < 0.05; uppercase letters: *p* < 0.01).

*A. protothecoides* and *S.liquefaciens* LZ03 were cultivated to the exponential growth phase and co-inoculated at algal:bacterial ratios of 5:1, 10:1, 15:1, and 20:1 (*v*/*v*) in fresh BG11 medium. The biomass and lutein content of *A. protothecoides* were monitored every 48 h. Figure 4a illustrates that all co-culture groups entered the logarithmic growth phase by day 2 and reached the stationary phase by day 8. In contrast, the axenic control culture initiated logarithmic growth on day 4 and reached the stationary phase on day 10. By the end of the cultivation period, cell density in all co-culture systems significantly surpassed that of the axenic control, indicating that co-cultivation with *S. liquefaciens* LZ03 enhances algal growth and accelerates the growth cycle. Analysis demonstrated that the 10:1 algal:bacterial ratio resulted in the highest final cell density (Figure 4b), suggesting that this ratio is optimal for maximizing *A. protothecoides* biomass production.

After 12 days of co-cultivation, *A. protothecoides* cells were collected through centrifugation for dry weight and lutein content determination (Figure 4b), within the scope of this experiment, algal-bacterial co-cultures at various medium-scale proportions significantly enhanced biomass (*p* < 0.01). The algal:bacterial ratio of 10:1 resulted in the highest biomass production (5.7 g/L, dry weight, DWt), marking a 28.3% increase compared to the pure algal culture (4.5 g/L, DWt). Analysis of lutein content showed a significant increase in all co-culture systems compared to *A. protothecoides* monoculture, indicating that co-cultivation with *S. liquefaciens* LZ03 not only enhances algal growth but also boosts lutein biosynthesis. The maximum lutein yield (7.8 mg/g, DWt) was observed at the 10:1 ratio, representing a 26% improvement over the control (6.2 mg/g, DWt).

### 2.5. Impact of Carbon Source on the Performance of A. protothecoides in Co-Culture with S. liquefaciens LZ03

**Figure 5:**
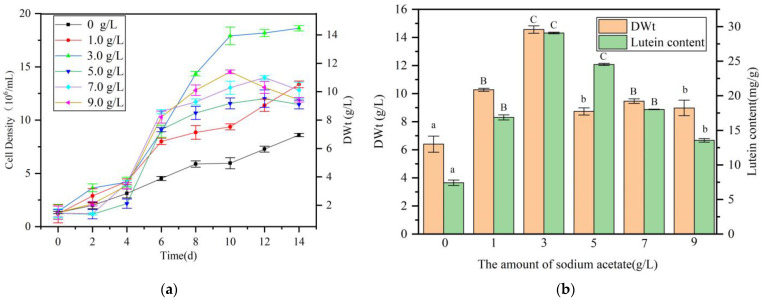
Effect of sodium acetate addition on lutein production in *A. protothecoides-S. liquefaciens* LZ03 co-culture. (**a**) Growth of *A. protothecoides* under co-culture conditions with different sodium acetate concentrations. (**b**) Biomass dry weight and lutein content in the co-culture system supplemented with different sodium acetate concentrations. Identical letters indicate no significant difference between groups (*p* > 0.05), while different letters signify significant differences (lowercase letters: *p* < 0.05; uppercase letters: *p* < 0.01).

The microalga *A. protothecoides* exhibits dual capabilities for photoautotrophic growth and heterotrophic cultivation with organic carbon sources. This study aimed to enhance lutein production by investigating the impact of organic carbon supplementation during algal-bacterial co-cultivation. Sodium acetate (2.0 g/L) and glucose (2.0 g/L) were added to BG11 medium, establishing co-cultures at a 10:1 algal-bacterial ratio. Results showed that the *A. protothecoides* culture supplemented with glucose perished by day 4 due to rapid *S. liquefaciens* LZ03 proliferation (Figure S1), utilizing glucose and producing metabolites toxic to the algae. Conversely, *A. protothecoides* supplemented with sodium acetate exhibited normal growth. Growth curves of *A. protothecoides* under co-culture conditions with varying sodium acetate concentrations are depicted in Figure 5a. Sodium acetate supplementation significantly enhanced algal growth compared to the control without sodium acetate, with the 3.0 g/L sodium acetate group displaying the highest growth rate. Post-cultivation, algal biomass from all groups was collected for dry weight and lutein content determination (Figure 5b). In the scope of this experiment, the addition of sodium acetate significantly increased the biomass of algal-bacterial co-cultures. When added at a concentration of 3.0 g/L, the maximum dry weight reached 14.6 g/L (DWt), which is 2.27 times that of the control group. Lutein content detection indicated that within, the addition of various concentrations of sodium acetate significantly increased the lutein content in algal cells. The highest lutein content was achieved when the culture medium was supplemented with 3.0 g/L of sodium acetate, reaching 29.1 mg/g (DWt), which is 3.9 times that of the control group.

### 2.6. Impact of Nitrogen Source Supplement on the Growth of A. protothecoides and the Accumulation of Lutein in Algal-Bacterial Co-Culture

**Figure 6:**
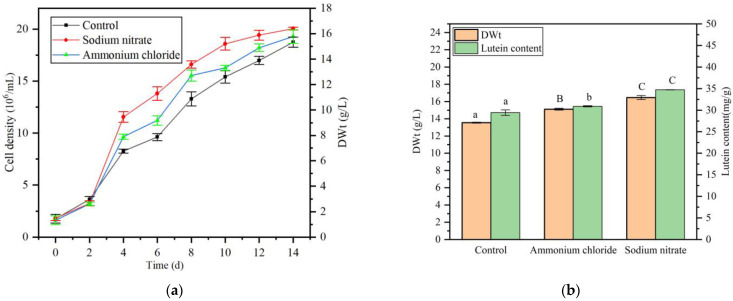
Effect of different nitrogen sources on *A. protothecoides* growth and lutein accumulation in co-culture with *S. liquefaciens* LZ03. (**a**) Growth curves of *A. protothecoides* in co-culture supplemented with different nitrogen sources. (**b**) Biomass dry weight and lutein content of *A. protothecoides* in co-culture supplemented with different nitrogen sources. (Data represent mean ± SD of three biological replicates. Statistical significance was analyzed by Student’s *t*-test.). Identical letters indicate no significant difference between groups (*p* > 0.05), while different letters signify significant differences (lowercase letters: *p* < 0.05; uppercase letters: *p* < 0.01).

To assess the influence of nitrogen sources, sodium nitrate (NaNO_3_) and ammonium chloride (NH_4_Cl) were added to BG11 medium at a concentration of 1.0 g/L. Analysis of *A. protothecoides* growth curves revealed that both ammonium and nitrate nitrogen significantly boosted algal growth in the co-culture system. Particularly, the stimulatory impact was more pronounced with nitrate nitrogen supplementation (Figure 6a). Subsequent determination of biomass dry weight post-cultivation showed a significant increase in the dry weight of *A. protothecoides* in co-culture with exogenous nitrogen supplementation. The highest dry weight (13.56 g/L, DWt) was recorded in the sodium nitrate-supplemented group, representing a 1.21-fold rise compared to the control group. Additionally, analysis of lutein content indicated enhanced lutein accumulation in *A. protothecoides* with nitrogen supplementation. Lutein content peaked at 34.73 mg/g (DWt) in the nitrate-supplemented group, which was 1.12 times higher than with ammonium chloride supplementation and 1.19 times higher than the control group. These findings suggest that nitrate nitrogen is more effective than ammonium nitrogen in increasing lutein production during the co-culture of *A. protothecoides* and *S. liquefaciens* LZ03 (Figure 6b).

**Figure 7:**
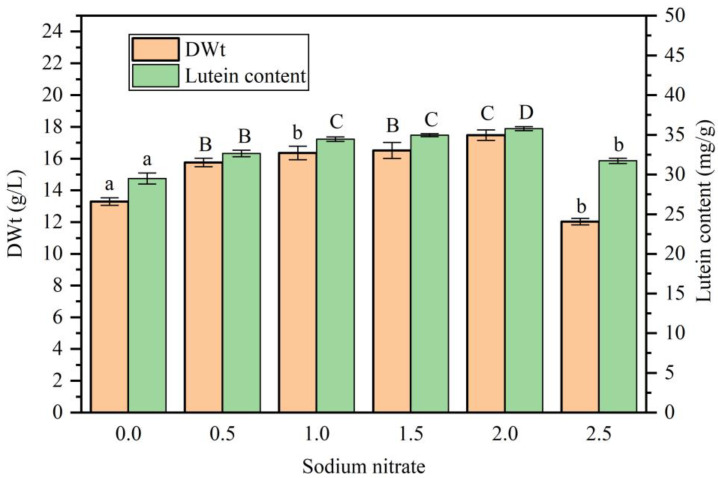
Effect of NaNO_3_ concentration on biomass dry weight and lutein content within the co-culture of *A. protothecoides* and *S. liquefaciens* LZ03. Identical letters indicate no significant difference between groups (*p* > 0.05), while different letters signify significant differences (lowercase letters: *p* < 0.05; uppercase letters: *p* < 0.01).

Subsequent studies further explored the impact of NaNO_3_ concentration on lutein production by *A. protothecoides* in co-culture with *S. liquefaciens* LZ03 (Figure 7). The addition of NaNO_3_ significantly affected cell growth. As the concentration of NaNO_3_ increased, the cell dry weight first increased and then decreased. The highest cell dry weight was 17.48 g/L (DWt) at a NaNO_3_ concentration of 2.0 g/L, which was 34.12% higher than that of the control group (*p* < 0.01). Similarly, the addition of NaNO_3_ also significantly increased the lutein content of algal cells. The highest lutein content was 35.78 mg/g (DWt) at a NaNO_3_ concentration of 2.0 g/L, which was 21.3% higher than that of the control group (*p* < 0.01).

### 2.7. Impact of N:P Ratio on the Growth of A. protothecoides and Lutein Content in Co-Culture with S. liquefaciens LZ03

**Figure 8:**
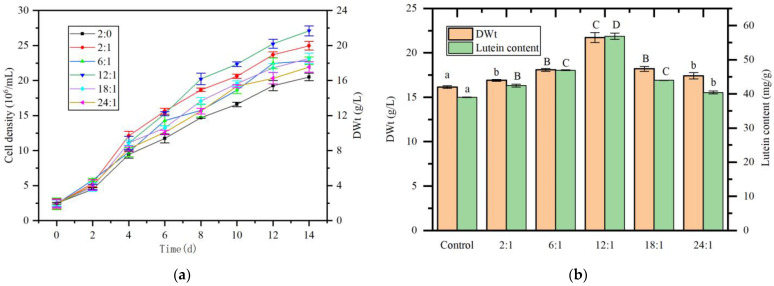
Effect of nitrogen-to-phosphorus (N:P) ratio on *A. protothecoides* growth and lutein accumulation in co-culture with *S. liquefaciens* LZ03. (**a**) Growth curves of *A. protothecoides* in co-culture supplemented with different N:P ratios. (**b**) Biomass dry weight and lutein content of *A. protothecoides* in co-culture supplemented with different N:P ratios. Identical letters indicate no significant difference between groups (*p* > 0.05), while different letters signify significant differences (lowercase letters: *p* < 0.05; uppercase letters: *p* < 0.01).

Figure 8a illustrates the impact of the nitrogen-to-phosphorus (N:P) ratio on the growth of *A. protothecoides* in the co-culture system. Across the tested ratios, the addition of sodium dihydrogen phosphate (NaH_2_PO_4_) at all N:P ratios promoted the growth of *A. protothecoides.* Particularly, the group with an N:P ratio of 12:1 demonstrated optimal growth, achieving the highest cell density at the end of the culture period. Analysis of cell dry weight revealed extremely significant between the experimental groups with N:P ratios of 6:1, 12:1, and 18:1 and the control group (*p* < 0.01), with the 12:1 group reaching the highest cell dry weight of 21.72 g/L (DWt), a 34.4% increase compared to the control group. Lutein content analysis indicated that, except for the 24:1 group which showed significant difference from the control group, all other experimental groups exhibited extremely significantly higher lutein content (*p* < 0.01). The group with an N:P ratio of 12:1 achieved the highest lutein content of 56.86 mg/g (DWt), representing a 45.7% increase compared to the control group (Figure 8b).

## 3. Discussion

### 3.1. Algal-Bacterial Co-Culture Significantly Enhances Microalgal Growth and Metabolite Accumulation

The integration of synthetic biology and green biomanufacturing through algal-bacterial co-culture technology has shown notable advantages in enhancing microalgal growth and metabolite synthesis efficiency. This approach exploits synergistic interactions among microorganisms to accelerate microalgal biomass accumulation and improve metabolite production. For example, co-culturing *Pseudomonas aeruginosa* with *C. vulgaris* for 22 days resulted in a microalgal biomass density of 1.483 × 10^7^ cells/mL, a 4.2-fold increase compared to the axenic *C. vulgaris* control (3.51 × 10^6^ cells/mL) [20]. Similarly, co-cultivation with a *Staphylococcus* sp. led to a 2.8-fold increase in *C. vulgaris* biomass compared to monoculture, underscoring the substantial biomass enhancement achievable through co-cultivation [21]. Apart from biomass gains, algal-bacterial co-culture not only enhances microalgal lipid content and quality but also activates secondary metabolic pathways through microbial interactions, facilitating the synthesis and accumulation of various high-value compounds. Wei et al. [22] demonstrated that co-culturing *C. vulgaris* with the diazotroph *Mesorhizobium* sp. increased total lipid content to 45.2% of dry weight (DWt) and neutral lipid content to 23% DWt, representing increases of 47.7% and 66.3%, respectively, compared to monoculture, along with a notable rise in the proportion of C18:1 (oleic acid). Furthermore, co-culturing *A. protothecoides* with *Rhodotorula glutinis* resulted in a 77% increase in total carotenoid content and shifted the proportion of astaxanthin from 3% to 12% [23].

### 3.2. The Precise Metabolic Division of Labor, Signal Communication, and Microenvironment Regulation Between Algae and Bacteria Enable the Growth of Microalgae and the Accumulation of Metabolites

The primary benefit of algal-bacterial co-culture systems stems from the intricate metabolic specialization and intricate signaling networks that develop between the microorganisms. These interactions not only address the metabolic constraints typical of monocultures but also boost overall system productivity by facilitating efficient nutrient cycling and energy transfer. For instance, in a co-culture involving the diazotroph *Mesorhizobium* sp., *C. vulgaris* supplies oxygen produced through photosynthesis for bacterial respiration, while the bacterium reciprocates by providing bioavailable nitrogen to the microalga [24]. This mutualistic relationship significantly alleviates nitrogen deficiency, which would otherwise hinder the growth of the microalga. Microbial signaling, characterized by the release of specific molecules to regulate collective behavior and adaptation to the environment, plays a crucial role in co-culture systems. Co-cultivating *Dunaliella salina* with epiphytic bacteria led to a 50% increase in β-carotene production, attributed to the presence of bacterial acyl-homoserine lactone signaling molecules [17]. This interaction also triggered the production of novel antimicrobial compounds. Furthermore, heterotrophic bacteria can promote microalgal cell division and indirectly enhance the levels of secondary metabolites such as lutein and zeaxanthin by generating hormones like indole-3-acetic acid (IAA) [25]. Additionally, algal-bacterial consortia improve microalgal resilience to extreme conditions by modulating the local microenvironment (e.g., pH, dissolved oxygen levels, osmotic pressure) [26].

### 3.3. The Limitations and Future Research Directions

This study is the initial documentation of *S. liquefaciens* LZ03, derived from activated sludge in a wastewater treatment plant, enhancing the growth of *A. protothecoides* and facilitating lutein accumulation in co-culture. While this study successfully employed a one-factor-at-a-time (OFAT) approach to optimize the co-culture conditions for *A. protothecoides* and *S. liquefaciens*, it is important to acknowledge the limitations of this methodology in comparison to statistical optimization techniques such as Response Surface Methodology (RSM) [27]. The primary limitation of the OFAT approach is its inability to efficiently identify and quantify potential interactive effects between critical variables (e.g., carbon source concentration, N:P ratio, and algal:bacterial inoculum ratio) [28]. Consequently, the conditions identified here, while significantly improved over the baseline, may not represent the absolute global optimum for the system. In contrast, RSM is specifically designed to model these complex interactions with fewer experimental runs, thereby revealing synergistic effects that might be missed by OFAT [29].

Building upon these findings, our future work will focus on implementing a more sophisticated statistical design. We plan to utilize the optimal levels identified in this study as the central points for a subsequent RSM experiment. A design such as the Central Composite Design (CCD) or Box–Behnken Design (BBD) will be employed to build a predictive model for lutein yield. This model will not only validate the current findings but is also expected to further refine the cultivation parameters by elucidating interaction effects, ultimately aiming to push lutein productivity closer to its theoretical maximum for this co-culture system. Furthermore, transcriptomic and metabolomic analyses are planned to unravel the underlying microbial interactions and metabolic mechanisms responsible for the enhanced lutein synthesis observed under optimized conditions.

Furthermore, to assess the robustness and scalability of the optimized process, future studies will specifically investigate batch-to-batch cultivation variability. Repetitive batch or semi-continuous cultivation modes will be implemented to evaluate the consistency of biomass productivity and lutein yield across multiple cycles. Understanding the sources of this variability is crucial for transitioning from lab-scale proof-of-concept to reliable industrial-scale production.

## 4. Materials and Methods

### 4.1. The Source and Cultivation of Algal Strains

The strain *A. protothecoides* was sourced from the Freshwater Algae Culture Collection at the Institute of Hydrobiology (Wuhan, China) and grown in BG11 medium under controlled conditions: temperature of 24 ± 1 °C, light intensity of 60 μmol photons m^−2^ s^−1^, and a 12-h light/12-h dark photoperiod.

### 4.2. Bacterial Isolation, Co-Culture, and Screening

Activated sludge obtained from a municipal wastewater treatment facility was suspended in double distilled H_2_O and subsequently subjected to a 10,000-fold serial dilution using sterile water. Portions of the diluted sample (100 μL each) were spread onto LB agar plates and then cultured at 28 °C for 24 h. Following the incubation period, the morphology of colonies was assessed, and individual colonies were chosen for inoculation into LB liquid medium and grown at 28 °C with agitation at 120 rpm for 12 h. Next, 10 mL of each bacterial culture was introduced into 100 mL cultures of *A. protothecoides* in the exponential growth phase. Co-cultivation was carried out for 10 days under the conditions outlined in Section 4.1. Subsequent to the co-cultivation period, the biomass of *A. protothecoides* and its lutein concentration were quantified. Bacterial strains that demonstrated a notable positive impact on either the growth of the algae or the accumulation of lutein were singled out for further investigation.

### 4.3. Biomass Quantification of A. protothecoides

Algal suspensions of different concentrations were created to construct a calibration curve. The optical density at 680 nm (OD_680_) was determined for each suspension. Subsequently, cells were harvested via centrifugation, rinsed twice with deionized water, and the resultant pellet was desiccated to a consistent weight at 80 °C. The DWt was documented, and a linear regression model relating DWt to OD_680_ was derived.

### 4.4. Detection of Lutein Content

A total of 0.1 g of algal powder was accurately weighed and 5 mL of methanol-dichloromethane was added (volume ratio 2:1). The mixture was homogenized twice at room temperature using a cell disruptor and then centrifuged at 6000× *g* for 4 min. The supernatant was collected and diluted to a final volume of 50 mL for subsequent analysis [30]. The entire extraction process was conducted under dim light and completed within 30 min. The lutein content in the extract was determined by Ultra-Performance Liquid Chromatography (Waters, Hong Kong) with modifications to the method of Sampathkumar et al. [31]. The chromatographic conditions were as follows:

Column: C18 column, 4.6 mm × 250 mm, 5 μm
Column temperature: 30 °C
Mobile phase: Methanol:water:acetonitrile = 10:10:80
Flow rate: 1.0 mL/min
Injection volume: 10 μL
Detection wavelength: 445 nm

Lutein standard (PhytoLab, Vestenbergsgreuth, Germany) was used to prepare a series of concentration standard solutions, and a standard curve of peak area versus lutein concentration was constructed using HPLC. The lutein content was calculated according to the following equation:

$$The\;lutein\;content=\frac{c*v*n}{m}$$

where *c* represents the concentration of lutein in the sample (mg/mL), *v* represents the volume of the extraction solution (mL), *n* represents the dilution factor, and *m* represents the mass of the algal powder (g).

### 4.5. Strain Characterization

The chosen bacterial strain underwent morphological characterization through Gram staining and molecular characterization following the protocol outlined by Machado et al. [32].

### 4.6. Bacterial Growth Curve Analysis

To determine the growth curve, a 1 mL sample of bacterial culture in exponential growth was transferred to fresh LB medium. The culture was then maintained at 28 °C with agitation at 180 rpm. Cell proliferation was tracked over 48 h by assessing optical density at 600 nm (OD_600_) every 2 h using a UV-Vis spectrophotometer (PE LAMBDA35, PerkinElmer‌, Shanghai, China). Growth curves were constructed utilizing Origin 9.0 software.

### 4.7. Analysis of Bacterial Growth in Co-Culture

*S. liquefaciens* was cultured to the logarithmic growth phase, harvested by centrifugation at 4000× *g* for 10 min, and washed twice with sterile distilled water. The pellet was resuspended in sterile BG11 liquid medium and adjusted to 10^6^ colony-forming units per milliliter (CFU/mL). An axenic culture of *A. protothecoides* was grown to 1 × 10^6^ cells mL^−1^. For co-culture, *S. liquefaciens* was inoculated at a volumetric alga-to-bacterium ratio of 10:1. Control flasks received an equivalent volume (10 mL) of sterile BG11 to equalize total volumes. Samples were taken at defined intervals, and the OD_600_ of the co-culture was measured against the axenic algal control as the blank to construct growth curves.

### 4.8. Effect of Bacterial Inoculum Size on A. protothecoides Growth and Lutein Accumulation

The bacterial strain, cultivated to the exponential growth phase, was harvested by centrifugation at 4000× *g* for 10 min, washed twice with sterile water, and resuspended in sterile BG11 liquid medium to a final concentration of 10^6^ CFU/mL. *A. protothecoides* was cultured to a cell density of 10^6^ cells/mL. Co-cultures were initiated by inoculating the algal culture with the prepared bacterial suspension at algal:bacterial inoculum ratios of 5:1, 10:1, 15:1, and 20:1 (*v*/*v*) in fresh BG11 medium. The cultures were maintained under the conditions outlined in Section 4.1, with biomass of *A. protothecoides* and lutein content assessed every 48 h. *A. protothecoides* cultured axenically (without bacterial inoculation) was employed as the control. The optimal algal:bacterial ratio for promoting algal growth and lutein accumulation was determined (All experiments were conducted in triplicate).

### 4.9. Effect of Carbon Source Addition on Lutein Production in the Algal-Bacterial Co-Culture

To assess the influence of carbon substrates on lutein production in a co-culture setting, sodium acetate (2.0 g/L) or glucose (2.0 g/L) was added to BG11 medium. The co-cultures were subsequently established at the optimal algal-to-bacterial ratio as identified in Section 4.8 and grown under standard conditions. The biomass of *A. protothecoides* and lutein levels were measured at 48-h intervals.

After identifying the optimal carbon source, its concentration was fine-tuned and incorporated into BG11 medium at varying concentrations (0, 1.0, 3.0, 5.0, 7.0, and 9.0 g/L). Co-cultures were then inoculated at the optimized algal:bacterial ratio as detailed in Section 4.8 and cultivated accordingly. A control group without added sodium acetate was also set up. The OD680 of the culture medium was measured at different time intervals to calculate the algal cell count and plot the growth curve.

### 4.10. Effect of Nitrogen Source on Lutein Production in the Algal-Bacterial Co-Culture

The BG11 medium, supplemented with the determined optimal concentration of the carbon source as outlined in Section 4.9, was modified by the addition of either 1.0 g/L sodium nitrate (NaNO_3_; serving as the nitrate nitrogen source) or 1.0 g/L ammonium chloride (NH_4_Cl; serving as the ammonium nitrogen source) to assess their distinct impacts on lutein production. Co-cultures were initiated at the optimal algal-to-bacterial ratio (as detailed in Section 4.6) and cultured under standard conditions, with quantification of *A. protothecoides* biomass and lutein levels conducted every 48 h.

After determining the optimal nitrogen source, its concentration was fine-tuned and incorporated into the carbon-optimized BG11 medium at different levels. Co-cultures were then initiated using the ideal algal-to-bacterial ratio as described in Section 4.6 and cultivated accordingly. Biomass of *A. protothecoides* and lutein content were assessed every 48 h, with all experiments conducted in triplicate.

### 4.11. Effect of Nitrogen-to-Phosphorus (N:P) Ratio on Lutein Production in the Algal-Bacterial Co-Culture

Expanding on the optimized concentrations of carbon and nitrogen sources as determined in Section 4.9 and Section 4.10, we investigated the impact of nitrogen-to-phosphorus (N:P) ratios on lutein yield. The BG11 medium was supplemented with potassium dihydrogen phosphate (KH_2_PO_4_) to achieve N:P ratios (by weight) of 2:0 (0 mg/L P, control), 2:1, 6:1, 12:1, 18:1, and 24:1. Co-cultures were inoculated at the optimal algal:bacterial ratio as specified in Section 4.6 and cultivated under standard conditions. Biomass of *A. protothecoides* and lutein content were quantified every 48 h, with all treatments conducted in triplicate.

### 4.12. Data Statistics and Analysis

In this study, each experimental group was conducted in triplicate. For each group, the mean and standard deviation of the three replicates were calculated to describe the central tendency and dispersion of the data. One-way analysis of variance combined with Dunnett’s multiple comparison test was used to analyze the significance of differences. The significance level was set at *p* < 0.05 for significant differences and *p* < 0.01 for highly significant differences. In the presentation of results, identical letters indicate no significant difference between groups (*p* > 0.05), while different letters signify significant differences (lowercase letters: *p* < 0.05; uppercase letters: *p* < 0.01).

## 5. Conclusions

The co-cultivation of algae with bacteria enhances microalgal growth efficiency and the synthesis of valuable compounds by modulating the algal microenvironment through processes such as carbon-nitrogen-phosphorus cycling, redox balance regulation, and intercellular signaling. This study reports the novel observation that co-culturing *A. protothecoides* with *S. liquefaciens* LZ03 significantly boosts both the growth and lutein accumulation in *A. protothecoides*. Through optimization experiments on inoculum ratio, exogenous carbon/nitrogen sources, and N:P ratio, it was determined that the highest biomass (21.72 g/L) and lutein content (56.86 mg/g, DWt) were achieved in BG11 medium supplemented with 3.0 g/L sodium acetate, 2.0 g/L sodium nitrate, and a N:P ratio of 12:1 maintained with sodium dihydrogen phosphate, with an algal-bacterial inoculum ratio of 10:1. Notably, the lutein yield in this optimized co-culture system was nine times higher than that in *A. protothecoides* mono-culture (6.2 mg/g, DWt), offering a promising strategy for sustainable industrial lutein production.

## Data Availability

The data that support the findings of this study are available from the corresponding author, upon reasonable request. This research was exempt from the requirement of ethics committee review or approval because it did not entail the utilization of patient information or the involvement of experimental animals.

## Supplementary Materials

The following supporting information can be downloaded at https://www.mdpi.com/article/10.3390/md23090360/s1. Figure S1: Growth of *A. protothecoides* in Glucose-Added BG11 Medium. A: Co-Culture of *A. protothecoides* and *S. liquefaciens* in Glucose-Added BG11 Medium. B: Growth of *A. protothecoides* monoculture in Glucose-Added BG11 Medium.
