# Supplementary material for: Co-Culture of Auxenochlorella protothecoides and Serratia liquefaciens Promotes Lutein Accumulation
Source: Mar Drugs. 2025 Sep 18;23(9):360. doi: 10.3390/md23090360 (PMC12471485; doi:10.3390/md23090360)
Supplement: Supplementary file 1 [file marinedrugs-23-00360-s001.zip › Figure S1.pdf]

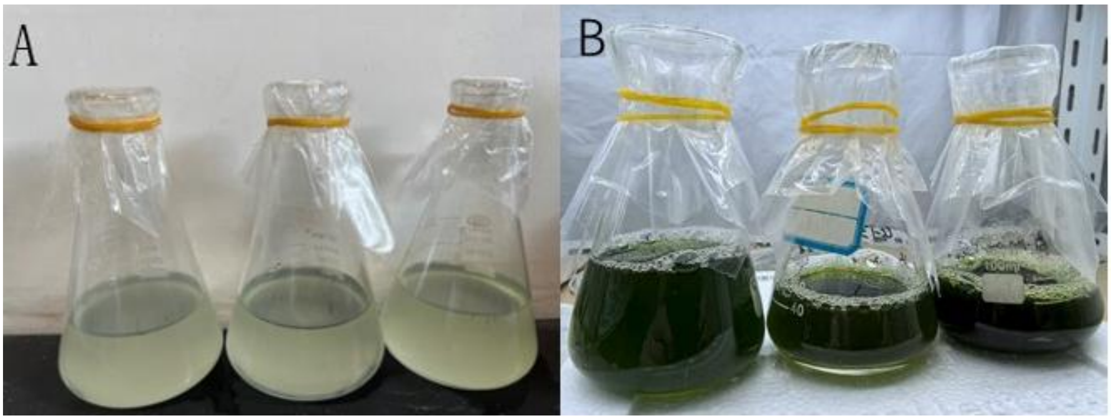

Figure S1. Growth of *A. protothecoides* in Glucose-Added BG11 Medium. A: Co-Culture of *A. protothecoides* and *S. liquefaciens* in Glucose-Added BG11 Medium. B: Growth of *A. protothecoides* monoculture in Glucose-Added BG11 Medium.
