# Supplementary material for: Co-Culture of Auxenochlorella protothecoides and Serratia liquefaciens Promotes Lutein Accumulation
Source: Mar Drugs. 2025 Sep 18;23(9):360. doi: 10.3390/md23090360 (PMC12471485; doi:10.3390/md23090360)

File: 0005_32724060600498_(9)_[27F].ab1 Run Ended: 2024-06-07 06:09:27 Signal G:2588 A:4024 T:2808 C:3311

Sample: 72703462546_32724060600498_(9)_[27F]_PY Lane: 4 Base spacing: 14.27 1276 bases in 16300 scans

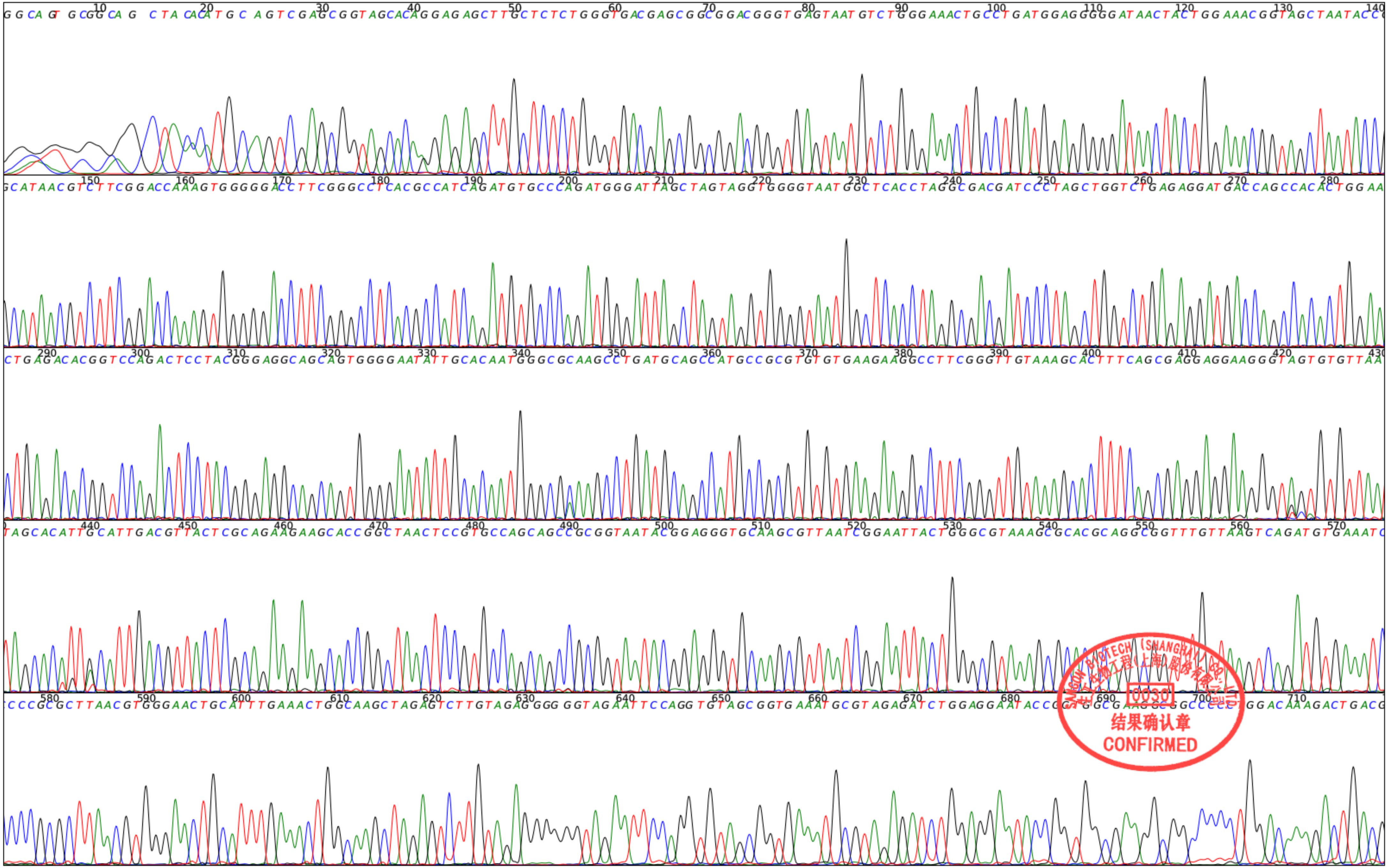

File: 0005_32724060600498_(9)_[27F].ab1 Run Ended: 2024-06-07 06:09:27 Signal G:2588 A:4024 T:2808 C:3311

Sample: 72703462546_32724060600498_(9)_[27F]_PY Lane: 4 Base spacing: 14.27 1276 bases in 16300 scans

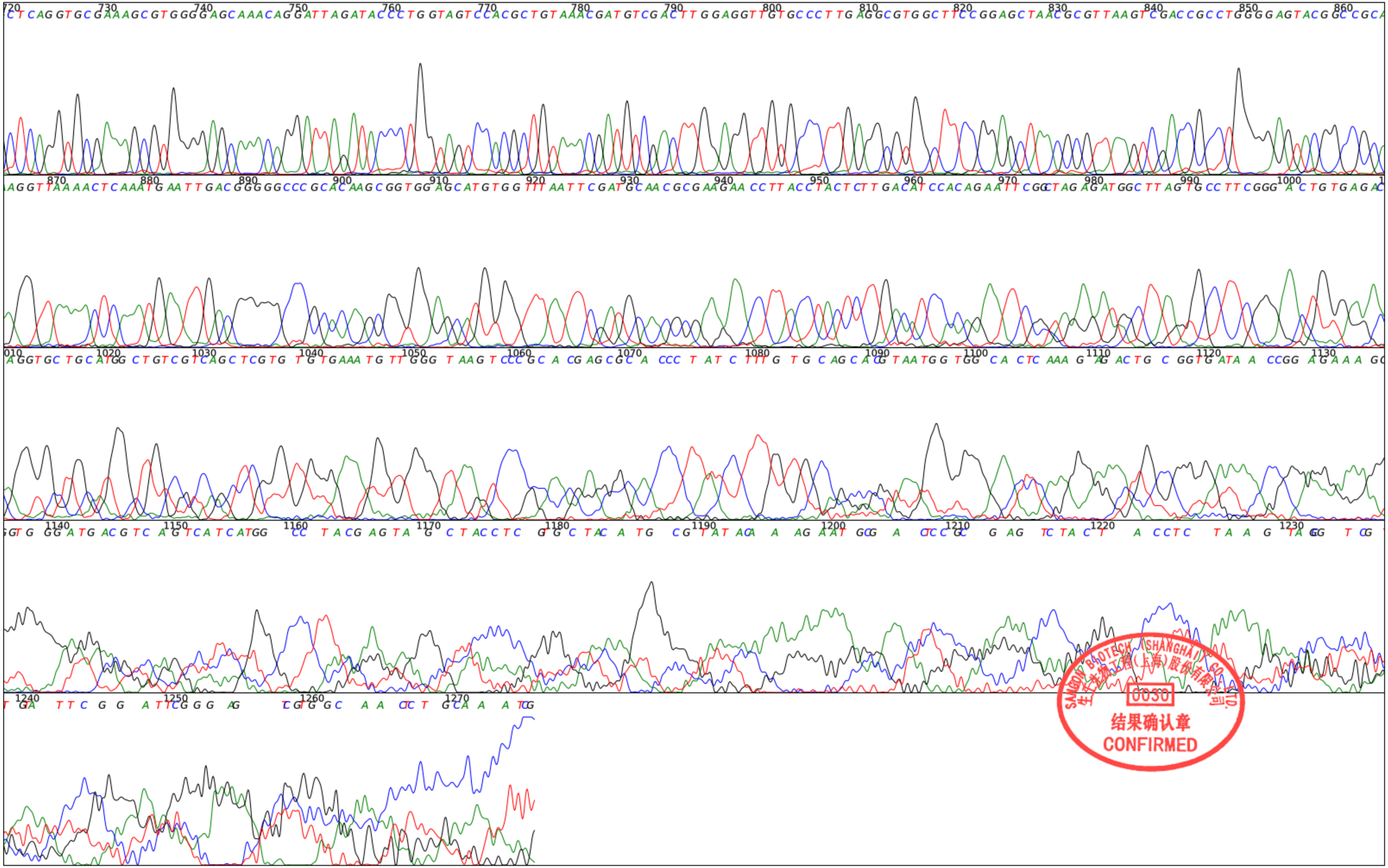

Supplement: Supplementary file 1 [file marinedrugs-23-00360-s001.zip › 16S DNA.zip!/0005_32724060600498_(9)_[27F]_H.pdf]
