# Supplementary material for: Co-Culture of Auxenochlorella protothecoides and Serratia liquefaciens Promotes Lutein Accumulation
Source: Mar Drugs. 2025 Sep 18;23(9):360. doi: 10.3390/md23090360 (PMC12471485; doi:10.3390/md23090360)

File: 0006_32724060600498_(9)_[1492R].ab1 Run Ended: 2024-06-07 06:09:27 Signal G:2694 A:3558 T:3783 C:4524

Sample: 72703462547_32724060600498_(9)_[1492R]_PY Lane: 4 Base spacing: 14.39 1260 bases in 16303 scans

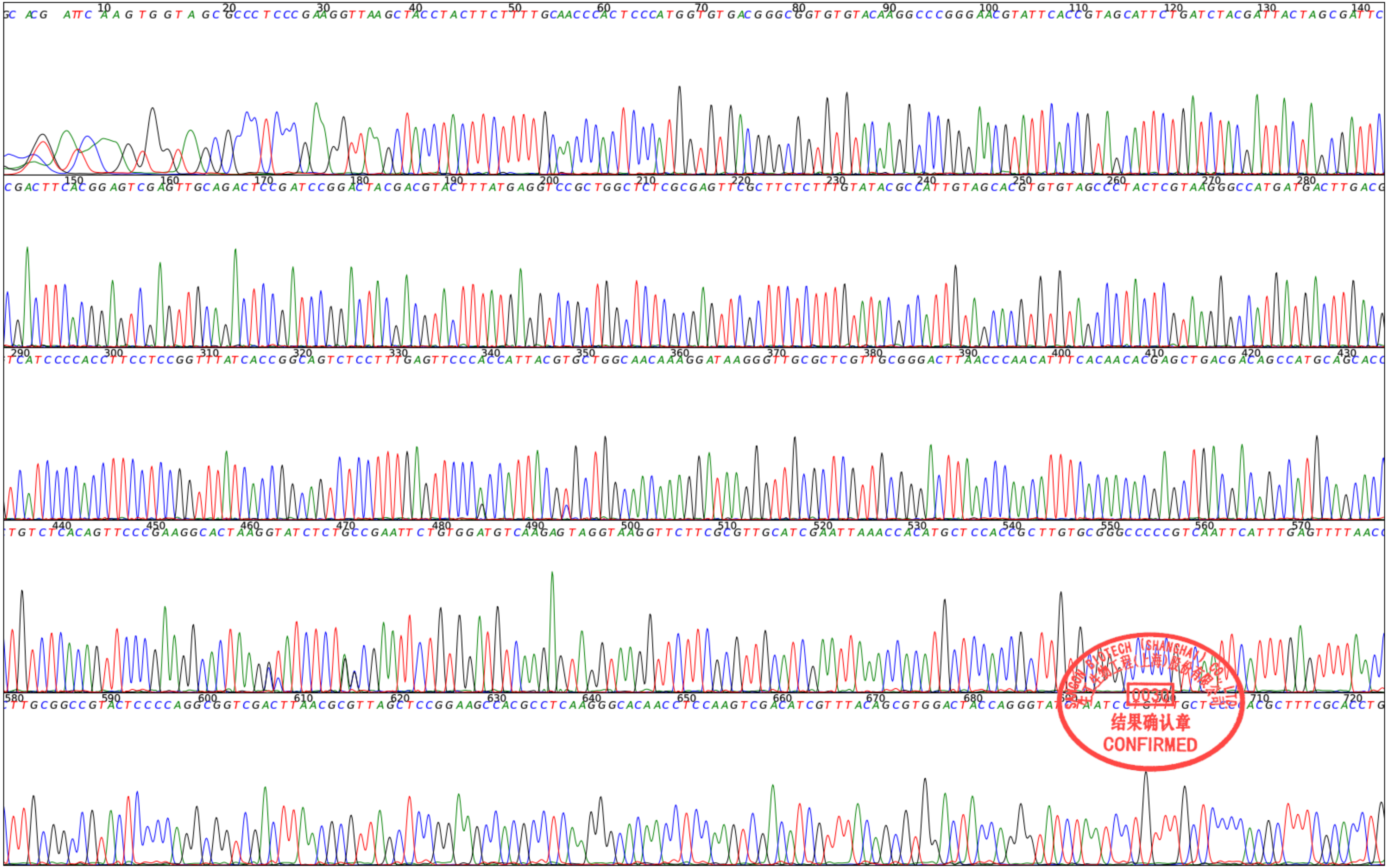

File: 0006_32724060600498_(9)_[1492R].ab1 Run Ended: 2024-06-07 06:09:27 Signal G:2694 A:3558 T:3783 C:4524

Sample: 72703462547_32724060600498_(9)_[1492R]_PY Lane: 4 Base spacing: 14.39 1260 bases in 16303 scans Page 2 of 2

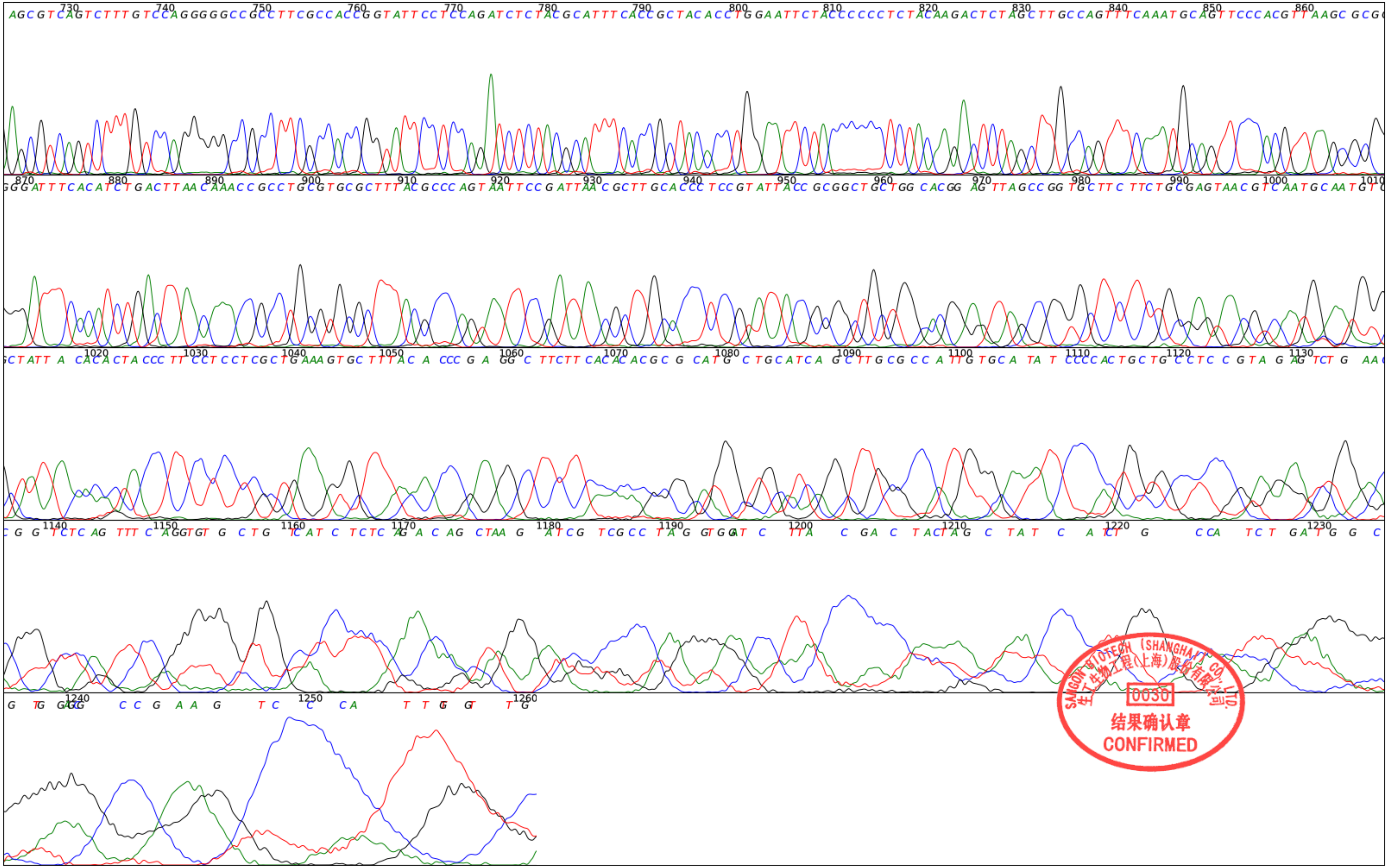

Supplement: Supplementary file 1 [file marinedrugs-23-00360-s001.zip › 16S DNA.zip!/0006_32724060600498_(9)_[1492R]_H.pdf]
